# How Does SUMO Participate in Spindle Organization?

**DOI:** 10.3390/cells8080801

**Published:** 2019-07-31

**Authors:** Ariane Abrieu, Dimitris Liakopoulos

**Affiliations:** CRBM, CNRS UMR5237, Université de Montpellier, 1919 route de Mende, 34090 Montpellier, France

**Keywords:** mitosis, spindle, microtubule-associated proteins, SUMO, SUMO-targeted ubiquitin ligases

## Abstract

The ubiquitin-like protein SUMO is a regulator involved in most cellular mechanisms. Recent studies have discovered new modes of function for this protein. Of particular interest is the ability of SUMO to organize proteins in larger assemblies, as well as the role of SUMO-dependent ubiquitylation in their disassembly. These mechanisms have been largely described in the context of DNA repair, transcriptional regulation, or signaling, while much less is known on how SUMO facilitates organization of microtubule-dependent processes during mitosis. Remarkably however, SUMO has been known for a long time to modify kinetochore proteins, while more recently, extensive proteomic screens have identified a large number of microtubule- and spindle-associated proteins that are SUMOylated. The aim of this review is to focus on the possible role of SUMOylation in organization of the spindle and kinetochore complexes. We summarize mitotic and microtubule/spindle-associated proteins that have been identified as SUMO conjugates and present examples regarding their regulation by SUMO. Moreover, we discuss the possible contribution of SUMOylation in organization of larger protein assemblies on the spindle, as well as the role of SUMO-targeted ubiquitylation in control of kinetochore assembly and function. Finally, we propose future directions regarding the study of SUMOylation in regulation of spindle organization and examine the potential of SUMO and SUMO-mediated degradation as target for antimitotic-based therapies.

## 1. The Biological Context

During mitosis, the microtubule (MT) cytoskeleton reorganizes to assemble the mitotic spindle that will capture chromosomes via their kinetochores and segregate them equally into two daughter cells. Compared to interphase, mitotic microtubules are shorter and more dynamic; they are nucleated from γ-tubulin complexes either at centrosomes or along pre-existing MTs [1]. Away from centrosomes, MT nucleation requires Ran-dependent liberation of spindle assembly factors such as TPX2, HURP, or NuSAP, together with augmin complexes that are assembled along preexisting microtubules [1,2,3,4,5]. At kinetochores, microtubules interact with several microtubule-associated proteins (MAPs, including microtubule-dependent motors), and appear as cold-resistant MT bundles called kinetochore-fibers (k-fibers) [6]. The latter are also stabilized along their length by microtubule connectors containing the scaffold protein clathrin, or the MAPs TACC3 and XMAP215 [7]. Interaction between kinetochores and microtubules is highly dynamic in order to correct erroneous attachment, and ensure chromosome bi-orientation before sister chromatids separate toward the two spindle poles [8]. This is achieved by a rapid turnover of phosphorylation/dephosphorylation events that are mainly catalyzed by Aurora-B kinase and the counteracting phosphatases [9]. Once chromosomes are separated, the spindle midzone is further bundled by MAPs, that crosslink microtubules thus becoming less dynamic [10].

**Figure 1:**
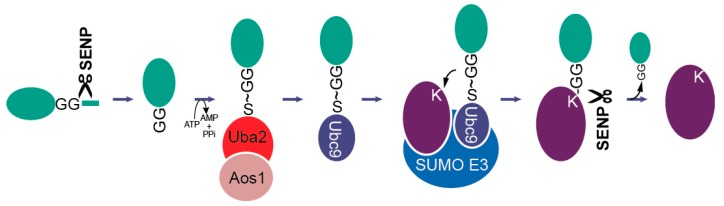
The SUMO pathway [13,18,19]. Similar to ubiquitylation, the SUMO conjugation pathway leads to formation of an isopeptide bond between the C-terminal carboxyl of the 11kDa protein SUMO and an ε-lysine of the target protein. SUMO precursors are first processed by SUMO isopeptidases, leaving a C-terminal di-glycine (GG) motif that is activated in an ATP-dependent manner, forming a thioester with catalytic cysteine of the heterodimeric E1-activating enzyme Uba2/Aos1. Next, SUMO is transferred onto the catalytic cysteine of the E2-conjugating enzyme (Ubc9) and finally onto the target protein, either directly or through a SUMO E3 ligase. SUMO itself can also serve as target for SUMOylation, leading to formation of polySUMO chains. SUMOylation is reversed by one of the SUMO isopeptidases that processed its precursor in the first step (SENP1-3 and SENP5-7 in human and Ulp1 and 2 in budding yeast). SENP1, 2, 3, and 5 belong to the Ulp1 branch, whereas SENP6 and 7 are more closely related to the Smt4/Ulp2 branch [20].

Control of MT dynamics and spindle organization during mitosis has long been known to be downstream of the kinase CDK1-Cyclin B that generates a phosphorylation cascade regulating the MAPs at play during mitosis [11]. This cascade culminates in metaphase and ends with the ubiquitin-dependent degradation of cyclin B and securin, a sister chromatid cohesion inhibitor [12]. While these two types of post-translational modifications (phosphorylation and ubiquitylation) have been extensively studied in this context, much less is known regarding modifications by the SUMO protein (Figure 1; for a review see [13]). This is indeed surprising, since SUMO has been first isolated in the context of spindle-related mechanisms either in Ran regulation or kinetochore function [14,15,16,17]. In addition, there is accumulating evidence that numerous mitotic motors, MAPs, and kinases are SUMOylated. The purpose of this review is to summarize information and discuss the possible molecular mechanisms involving SUMO in mitotic spindle organization.

Contrary to ubiquitylation, SUMOylation does not always require a E3 ligase, at least in vitro [21]. Nevertheless, three classes of E3 ligases are the best characterized (for a review, see [22]; other types of SUMO E3s also exist). The SP-RING family was identified with Siz1/Siz2 in budding yeast that share a conserved RING-related motif with PIAS (protein inhibitor of activated STAT) [23], responsible for the interaction of the SUMO E3 with Ubc9 [24].

The second class of SUMO E3s is the Ran-binding protein 2 (RanBP2), which is a large (358 kDa) component of the nuclear pore complex (NPC). This multidomain E3 ligase contains two internal repeats (IR) displaying the E3 ligase domain IR1-M-IR2 [25,26,27]. The IR-domain is also the site of assembly of the RRSU complex that contains the SUMO1-modified form of RanGAP1 (RanGAP1•SUMO1) and Ubc9 [28]. This complex is recruited to the outermost part of the kinetochore where it co-localizes with KIF10/CENPE [29].

The founding member of the third class of SUMO E3s is ZNF451 that is highly specific for SUMO2/3 [22]. Family members possess an internal PxRP motif surrounded by 2 SIMs. No spindle function has yet been uncovered for this less characterized family of E3 ligases.

## 2. SUMO Targets

### 2.1. SUMOylation Concerns One-Third of Human Proteins and Spindle Proteins are not an Exception

*Saccharomyces cerevisiae* contains a single SUMO protein, Smt3 (suppressor of Mif2 [16]), while mammalian cells express up to five SUMO paralogs (SUMO1-5). SUMO2 and SUMO3 being almost identical (97% in human), they are usually referred to as SUMO2/3. SUMO1 is the only other ubiquitous homolog, whereas expression of SUMO4 and SUMO5 is restricted to a subset of tissues [30,31]. The yeast Smt3 is essential, while SUMO1 is not. SUMO2 gene knock-out is lethal around embryonic day E10.5, suggesting that SUMO2 perform essential function despite its high redundancy with SUMO3 [32]. Deletion of the gene encoding the unique E2 enzyme Ubc9, is lethal in both yeast and mammals (mice die early in development, prior to E7.5), demonstrating that SUMOylation is essential [33].

Under standard growth conditions, SUMOylation is quite promiscuous since only 28% of the substrates follow the minimal SUMO consensus motif (SCM) ΨKxE, where Ψ is an hydrophobic residue [34]. The second most frequent sequence is the reverse SCM E/DxK, but the presence of the acidic glutamate (E) can also be spread within the −15/+15 positions surrounding the SUMOylated lysine [34]. On top of that, the presence of an acidic glutamate residue is efficiently mimicked by phosphorylated serine or threonine. Altogether, there is not an absolutely reliable way to identify SUMOylation sites a priori.

Intriguingly, only a small percentage of a given SUMO substrate is usually SUMOylated, exceptions taken apart [14]. Many new SUMO conjugates have been identified recently due to the increasing sensitivity of mass spectrometry and low-level SUMOylation of these proteins could be unspecific, without any regulatory function. Against this idea is the fact that abrogation of SUMOylation of even low-level SUMOylated proteins very often leads to a measurable phenotype. For example, only a small percent of Ndc10 and Cep3 is SUMOylated in vivo, however their non-SUMOylated variants do not localize to the spindle midzone [35,36]. It has been proposed that the conjugation–deconjugation cycle is continuously ongoing, ensuring a high SUMOylation turnover, although the steady-state amount of SUMOylated forms is low at any moment [37].

SUMO probably competes with acetylation, ubiquitylation, and methylation for target lysines. Interestingly, a proteome study revealed that lysines residing in disordered regions are preferentially targeted by SUMO and not by the other PTMs [34]. This whole-cell study also revealed that SUMOylation is very frequent: One-third of all human proteins (6747 proteins) display SUMO2 sites [34]. The majority (2/3) of SUMOylated proteins have at least two SUMO sites, while 1000 harbor more than 10, and 64 targets are SUMOylated on more than 40 lysines. Among the 10–40 SUMO sites category, one finds many mitotic proteins, such as the spindle MAP TPX2, the kinesins KIF4, KIF18A, KIF22, and KIF23, the centromeric protein CENPC, the centrosomal protein CEP57, and the kinases CDK1, Aurora B/AURKB, and TTK/Mps1. Strikingly, an average of 18% of all lysines were SUMOylated in SUMO target proteins, which reveals an unsuspected abundance of SUMO modifications, and again highlights the difficulty to generate SUMO-free mutants. A more recent study identified additional sites [38].

Numerous SUMOylation events were found proximal to CDK-dependent phosphorylation, which points to a role for SUMO during mitosis, when CDK activity is at its highest [39]. However, a similar number of SUMOylation sites become upregulated or downregulated after CDK inhibition suggesting a dynamic regulation of SUMO at the G2/M and metaphase/anaphase transition.

### 2.2. Centromere/Kinetochore and Chromosome Associated Proteins

**Figure 2:**
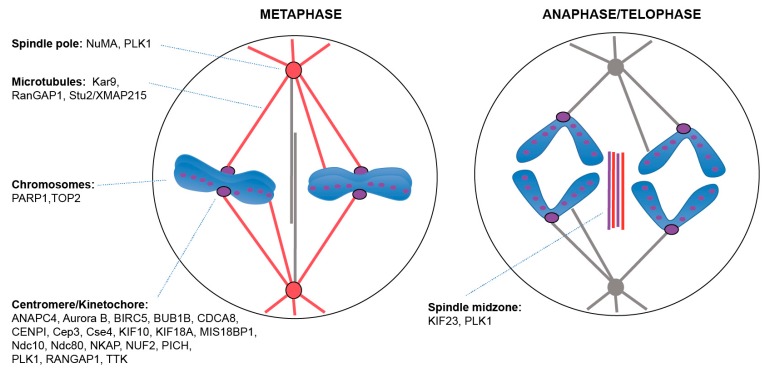
SUMO targets on the mitotic spindle in metaphase (left) and anaphase/telophase (right). In metaphase, SUMO1 (colored in red) mainly localizes around spindle poles and MTs while SUMO2/3 (colored in purple) is enriched at centromeres/kinetochores and around chromosomes. After the metaphase/anaphase transition, SUMO1 relocalizes to the spindle midzone, and SUMO2/3 remains around chromosomes while also localizing to the spindle midzone [36,74]. SUMOylated targets are indicated at the different mitotic locations. Shown are proteins whose SUMOylation has been studied during mitosis.

A striking majority of the proteins whose SUMOylation has been studied during mitosis (Table 1, Figure 2) localizes at the centromere/kinetochore. Among these are centromeric proteins Cse4, Cep3, Ndc10 in yeast, and CENPI [35,40,41], the helicase PICH [42], and the centromere-associated kinase Aurora B/AURKB along with its partners Borealin/CDCA8, MIS18BP1 and Survivin/BIRC5 (Bir1 in *S. cerevisiae*) in mammalian cells [35,43,44,45,46]. At the level of the outer kinetochore, one finds SUMOylated Ndc80 and NUF2 [35,36], as well as the kinesins CENPE/KIF10 [36] and KIF18A [47], the RNA binding protein NKAP [48], the Ran GTPase activating protein RanGAP1 [49], the ANAPC4 subunit of the APC/C [50,51], along with the protein kinases BUB1B/BubR1 [52] and MPS1/TTK [53]. When known, most of these proteins are SUMOylated by SUMO2/3, and often require PIAS4 (also known as PIASγ) as a E3 SUMO ligase (see Table 1). Beyond the centromere/kinetochore, the whole chromosome also contains SUMOylated substrates such as the Poly-ADP Ribose Polymerase PARP1 [42,54] and the Topoisomerase II TOP2 [55,56,57,58,59]. Their SUMOylation also depends upon SUMO2/3 and the E3 SUMO-protein ligase PIAS4.

### 2.3. Spindle-Associated Proteins

At the spindle pole, studied SUMOylated substrates are PLK1 [73,79] and the MAP NuMA [71]. In contrast to chromosome-associated SUMO substrates, it is rather SUMO1 that modifies PLK1 and NuMA (Figure 2). This is in accordance with the observed presence of SUMO1 at spindle poles and SUMO2/3 at chromosomes/kinetochores [36]. On the spindle, the yeast MAP protein Stu2/XMAP215 was identified as SUMO conjugate [77], as well as the yeast spindle positioning factor Kar9 [67,80]. The number of SUMOylated spindle proteins will very likely increase, since several MAPs are found to be SUMOylated within the SUMOylome proteomic data (see §4 and Table 2).

Less is known regarding SUMOylated targets after the metaphase/anaphase transition (Figure 2). PLK1 was shown to colocalize with SUMO1 and SUMO2/3 at the spindle midzone in telophase in mice meiosis [73]. Later on, septins localize to the midbody, where septin bundling activity requires their SUMOylation, which is critical to cytokinesis in human cells [75,76,81,82].

## 3. SUMO Functions in Spindle Organization

### 3.1. SUMO and SUMO Interacting Motifs (SIMs)

Relatively little is known on the functional consequences of SUMOylation regarding the aforementioned, spindle-associated proteins. Conjugation of SUMO to a protein occupies a lysine residue and creates an additional protein surface on the substrate. Moreover, SUMO can compete with other lysine-targeted PTMs but also inhibit or promote intra- and intermolecular interactions. Perhaps the most interesting outcome of SUMOylation is to promote protein-protein interactions via its binding to SUMO interacting motifs (SIMs).

SIMs are classified in three categories: a class I SIM is defined by a short stretch of three to four hydrophobic amino acids embedded in a beta-strand that is flanked by acidic amino acids interacting with basic amino acids on SUMO surface [83,84]. These SUMO-SIM interactions are of very low affinity (1-100 uM) and are well characterized (reviewed in [85]). A second independent domain (class II SIM) is responsible for interacting with Ubc9 (far from the thioester bond between Ubc9 and SUMO). This non-covalent interaction is essential to promote polySUMO chain formation [86] and is of much higher affinity than the SUMO-class I SIM interaction (82 nM). Class I and II SIMs do not compete, since they interact with SUMO on opposite binding surfaces. In between these two domains, one finds another independent class III interaction surface that has been initially described to interact with the ubiquitin E3 ligase HERC2 via SUMO1 ZZ zinc finger domain [87]. SIM motifs can be predicted to some extent (http://sumosp.biocuckoo.org/online.php, http://www.jassa.fr/, http://predictor.nchu.edu.tw/SUMOgo/). It is generally thought that SUMO-SIM interactions undergo rapid turnover, but quantitative in vivo measurements are lacking.

### 3.2. SUMO Functions: Direct and Indirect Regulation of Protein Localization within the Mitotic Spindle

Similar to the ubiquitin-interacting domains, SIM domains have dramatically increased the repertoire of possible SUMO functions regarding organization of protein complexes. Indeed, although 30–50% of the mitotic SUMOylated proteins/complexes that have been studied so far are enzymes, SUMOylation seems to rarely affect their activity directly (Table 1). When described, such as for Aurora B, PICH, PARP1 and Topoisomerase II, modulation of their enzymatic activities by SUMOylation is rather mild or controversial [42,44,45,55,56]. Regarding direct control of localization by SUMOylation at the kinetochore, only Cse4, CENPI, Aurora B, Ndc10, KIF18A and RANGAP1 are concerned (see Table 1). Even in this case, the molecular mechanism very likely involves interaction of the SUMOylated factors with SIM-containing proteins at the kinetochore.

In fact, SUMOylation can more often indirectly control localization of proteins bearing SIM domains. For instance, although CENPE is itself SUMOylated, it is surprisingly its interaction with SUMOylated NKAP that recruits it to the kinetochore [48]. Indeed, CENPE SIMs are required for its kinetochore localization [36]. While NKAP SUMOylation is essential for CENPE recruitment to the kinetochore, it is BUB3 that promotes NKAP recruitment [48]. Altogether, this data suggests that SUMO can control protein localization to kinetochores via SUMO-SIM interactions, and this may affect also proteins that are not SUMOylated per-se.

### 3.3. SUMO Functions: Group SUMOylation and Phase Transition

Polyvalent interactions between SUMO and SIM can also give rise to larger protein-assemblies, a process well documented for SUMO in the context of DNA repair (group SUMOylation) [88]. These multiple SUMO-SIM interactions that foster intermolecular interactions have been referred to as “SUMO glue”. Indeed, the low affinity SUMO-SIM interaction (1–100 µM range) can turn into extremely high affinity “glue” (<1 nM range) within a group of proteins interacting via multiple SUMO-SIM interactions [89].

**Figure 3:**
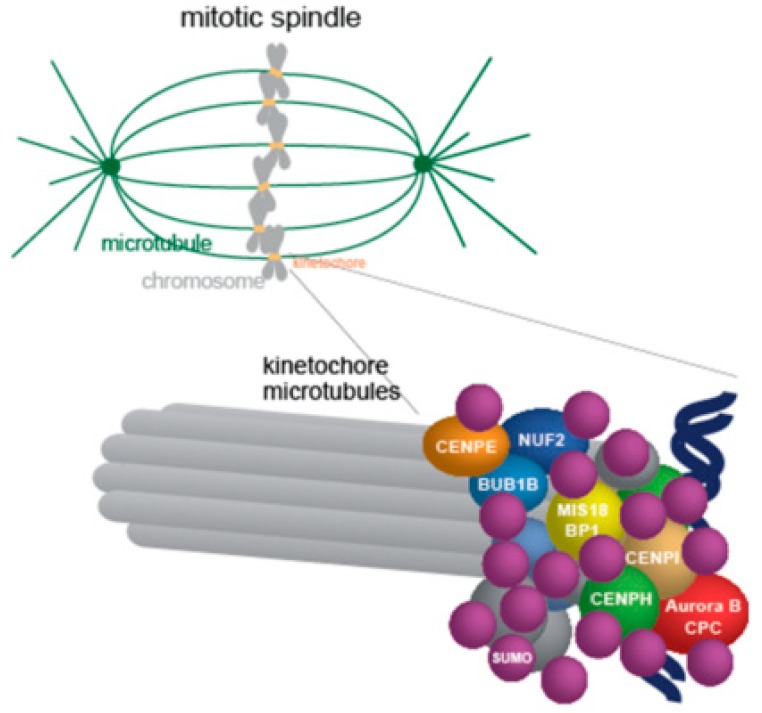
The kinetochore may contain group SUMOylated subcomplexes. A large number of kinetochore proteins have been identified as SUMO conjugates (see Table 2). Specific subcomplexes may be present as group SUMOylated complexes stabilized through SUMO– SUMO interacting motifs (SIM) interactions between their members. Shown here are the studied cases: The mammalian CENPH/I/K complex, MIS18BP1, CENPE, NUF2, and BUB1B/BuBR1 or the Aurora B-CPC complex (CPC, Survivin-Borealin-INCENP, for references see Table 1), but many more may exist (see §4).

Is group SUMOylation a mode that applies on the mitotic spindle? This could be likely the case for kinetochores in view of the large number of SUMOylated kinetochore proteins. The localization defects of kinetochore proteins mentioned in the previous paragraph may be well explained in the context of group SUMOylation. It is also conceivable that kinetochores consist of group SUMOylated subcomplexes stabilized through SUMO-SIM interactions (Figure 3). Among them the mammalian CENP/I/H/K, CENPC/MIS18BP1, the yeast Ndc10/CBF3 complex, the Ndc80/NUF2 complex (yeast, mammalian) and the Aurora-B CPC complex are the most obvious candidates, [35,40,63,70].

A nice example for a SUMO-SIM assembly is the Ring Complex required for meiotic chromosome congression in *C. elegans* oocytes [68]. In this structure that localizes between homologous chromosomes, the GEI-17/PIAS E3 SUMO ligase SUMOylates itself, kinesin 4/KLP-19 and probably other factors. In a feedback mechanism, GEI-17 also recruits its SUMOylated targets via SIM motifs that are also present on checkpoint protein BUB-1/BUB1 [90]. As the Aurora B-CPC complex, that can also be SUMOylated by GEI-17, localizes, together with the SENP ULP-1 to the Ring Complex [62], the latter represents probably a dynamic group SUMOylated signaling hub on the meiotic spindle. It is tempting to speculate that the RanBP2 ligase in the RRSU complex forms a similar group-SUMOylated complex at kinetochores, since its downregulation leads to mislocalization of a number of kinetochore factors (i.e., RANGAP1, CENPE, CENPF [49]).

Group SUMOylated complexes on DNA repair foci may represent biomolecular condensates that form a separate phase from their surroundings. In part, this might be due to the polyvalent SUMO-SIM interactions between proteins participating in the DNA repair process [89,91,92]. PolySUMO-polySIM interactions have been shown to phase separate in vitro [89] and lysines in disordered protein regions (that are one of the underlying features promoting phase separation) are preferentially SUMOylated [34]. It remains to be seen whether this type of phenomenon applies for in vivo SUMOylated spindle complexes at kinetochores or at spindle poles. Alternatively, SUMOylation may be a means to regulate assembly and disassembly of condensates, but not the basis for their formation.

Whether phase-separated or not, the question arises, what distinguishes the different SUMO-SIM groups. In the case of the mitotic spindle it is quite striking to note that SUMO2/3 is enriched around chromosomes and kinetochores, while SUMO1 is mainly found close to microtubules, but particularly enriched at spindle poles [36,73]. Proteins at these sites may contain different types of SIM motifs that distinguish between SUMO isoforms [93]. The finding that K37 SUMO1 acetylation (K33 in SUMO2) neutralizes the basic charge interacting with the SIM acidic amino acid [94] could provide another explanation. SUMO acetylation is translated to decreased SUMO-SIM affinity for numerous proteins. Whether there is increased SUMO acetylation in the vicinity of the chromosomes or elsewhere around the spindle remains to be determined. Finally, in the context of phase separation, the composition of kinetochore SUMO-SIM groups could lead to condensates with properties that are distinct from other distant SUMO-SIM containing phases (for a review about biomolecular condensates, see [95]).

### 3.4. SUMO as a Signal for Degradation of Spindle Proteins: SUMO-targeted Ubiquitin Ligases

SUMO can be attached to one lysine (mono-SUMOylation), multiple lysines (multi-SUMOylation) or form a SUMO chain onto a target lysine (poly-SUMOylation) [96]. SUMO chains are assembled via an N-terminal lysine (K7 for SUMO1, K11 for SUMO2/3 and K11/K15/K19 in SMT3). Large scale proteomic studies revealed that under standard growth conditions, SUMO2/3 is more prone to chain formation (>90%) than SUMO1 in cellulo [34]. SUMO chains are able to recruit SUMO-targeted ubiquitin ligases (STUbLs) like RNF4 (Ring Finger Protein 4, Slx5/Slx8 heterodimer in *S. cerevisiae*) and RNF11/Arkadia, a class of ubiquitin E3 enzymes that ubiquitylate SUMOylated proteins [97]. The STUbLs bear a RING domain that is important for dimerization and ubiquitin ligase activity, and multiple SIMs that determine the specificity for SUMO chains [98]. STUbL-mediated ubiquitylation can lead to proteasomal degradation [99,100,101], but also serve as non-degradation signal, linked to formation of K63 ubiquitin chains [102]. Of note, STUbLs target also many enzymes of the SUMO machinery for degradation, affecting the overall SUMO equilibrium in cells [103,104].

As mentioned before, SUMO-SIM interactions may stabilize the entire protein assemblies as group-SUMOylated complexes. Herein, the interplay between SUMOylation by SUMO E3s and de-SUMOylation by SENPs may regulate complex dynamics through removal of factors enabling other proteins to incorporate into the assembly. An alternative to deSUMOylation is degradation or regulation of SUMOylated proteins through SUMO-targeted ubiquitylation [40,63].

**Figure 4:**
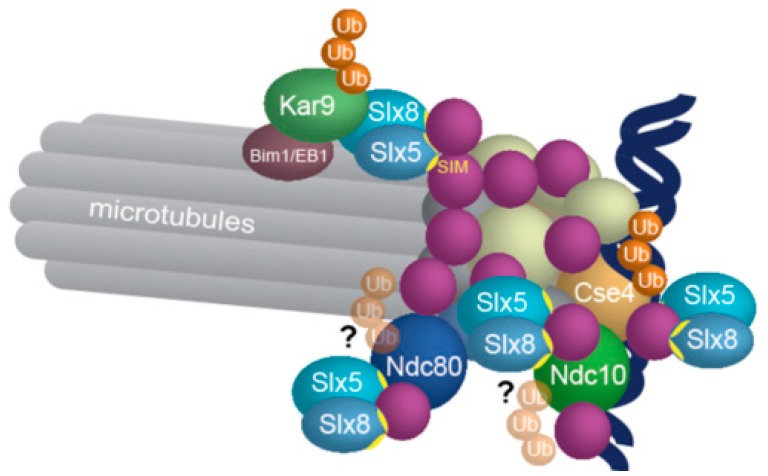
STUbL-dependent ubiquitylation may act as a quality control during kinetochore assembly. RNF4 has been shown to antagonize SENP6 and ubiquitylate the centromeric proteins CENPI, CENPH, and MIS18BP1, factors that are required for loading of CENPA onto centromeres. In addition, yeast Slx5/RNF4 ubiquitylates the centromeric histone Cse4/CENPA for degradation and interacts in yeast 2-hybrid with the inner kinetochore factors Ndc10 and Cep3. STUbLs may also eliminate mislocalized MAPs. Kar9, present normally only on astral microtubule plus-ends, is targeted for degradation by yeast STUbLs when it reaches the plus-ends of kinetochore microtubules. Shown here are the yeast proteins; for references, see text and Table 1.

In *S. cerevisiae*, *slx5∆* and *slx8∆* deletion mutants show severe mitotic defects such as aneuploidy and aberrant spindle formation and positioning [105]. Deletion of the human orthologue RNF4 also causes chromosome segregation errors [105,106], suggesting that the STUbL mitotic function is conserved. In fact, interplay of SUMOylation and SUMO-targeted ubiquitylation/degradation could regulate assembly of kinetochores and associated complexes (Figure 4).

In support of this idea, localization and abundance of the protein CENPI on kinetochores is regulated through competition between SUMOylation and SUMO-mediated ubiquitylation by the deSUMOylase SENP6 and RNF4, respectively [63], and the same seems to apply for the CENPA loading factor MIS18BP1. The latter was identified in a proteomic screen of SUMOylated factors that are co-modified by SUMO and ubiquitin. In addition, MIS18BP1 interacts with RNF4, and RNF4 inhibition leads to reduced ubiquitylation of the protein and accumulation of its SUMOylated forms. Lastly, MIS18BP1 becomes degraded after depletion of SENP6 and recovers after proteasomal inhibition by MG132 [70].

Another example is activity of the yeast Aurora B-CPC complex that also seems to be regulated by SUMO-dependent degradation at kinetochores [107]. Upon mild replication stress, STUbLs ubiquitylate the CPC proteins Sli15/INCENP and Bir1/Survivin and target them for proteasomal degradation. As a consequence, cells inactivate the spindle assembly checkpoint and are able to adapt and proceed into anaphase after mild replication stress.

In a third example, SUMO and STUbLs seem to be involved in mobilization of proteins from the yeast kinetochore to the spindle midzone. Non-SUMOylated variants of yeast Ndc10 and Bir1/survivin fail to translocate to the midzone. 

Finally, another possibility is that SUMO-targeted ubiquitylation acts as a quality control pathway (Figure 4). STUbLs ubiquitylate the yeast centromeric histone Cse4 along with three other ubiquitin E3s [64,65,108,109] and prevent Cse4 localization to euchromatin [64]. Similarly, STUbLs may function to remove unwanted or misfolded proteins form kinetochores. Evidence for this came from the surprising finding that the Kar9 protein, a yeast factor that localizes to microtubule plus-ends of cytoplasmic microtubules only, is ubiquitylated by Slx5/Slx8 at kinetochores and subsequently degraded by the proteasome [66]. One could speculate that STUbLs eliminate a protein that is at the wrong (the kinetochore) microtubule plus-ends. Whether STUbLs generally regulate mislocalized MAPs or misfolded kinetochore factors is however an open question.

## 4. Many More Mitotic Proteins are SUMOylated–How to Study Them?

Surprisingly, the mitotic proteins whose SUMOylation has been studied (Table 1) are not necessarily also the most abundantly SUMOylated (see Table 2). Numerous paradigms exist: for instance, it is noteworthy that KNL1 that was already known to perform scaffolding function [110] at the kinetochore can bear up to 87 SUMOylation sites! This suggests that KNL1 could stabilize the interaction with kinetochore partners via SUMO-SIM interactions. The same logic could apply for KNL2 at the centromere, since this protein was identified with no less than 80 SUMOylation sites. In the spindle area, it is TPX2 with 39 reported SUMOylation sites that could stabilize interaction with its many partners. CENP-C has a central role in recruiting the kinetochore onto the centromere [111], that could be favored by its 28 SUMOylation sites. From the many kinesins that are SUMOylated, the chromokinesins KIF22 and KIF4A are the most heavily modified. Although it is tempting to speculate that such an extensive SUMOylation might fulfill some important function, SUMOylation can also be essential for localization or activity of protein/complexes that bear only one or two SUMO, such as ANAPC4 [50,51] or CENP-I [40,63]. In between these extremes, there are even more recent examples of mitotic proteins for which the function of SUMOylation remains unknown (Table 2).

The caveat with the study of multi- and poly-SUMOylated proteins or group SUMOylated complexes lies in the difficulty of obtaining loss of function mutants because a) several dozens of lysines can be modified and/or SUMOylation can pass on to another lysine residue upon mutation of the acceptor lysine, b) the SUMO-associated function can arise not from direct SUMOylation, but from non-covalent interaction with SUMO via a SIM domain, and c) within group SUMOylated complexes, simultaneous abrogation of a critical number of SUMO-SIM interactions may be required in order to obtain an effect on the complex [91], and this is more so for assemblies that phase-separate. In all these cases, the use of SUMO mutants that allow to universally reduce SUMOylation provides a useful approach with severe limitations, due to the pleiotropic effects of these mutants and the same is true for approaches that would abrogate SUMO-SIM interactions (SUMO-SIM interaction inhibitors or specific SUMO variants [112]). Another possibility is reconstitution of these complexes in vitro, but this remains an extremely challenging undertaking. 

“Gain of SUMOylation” approaches are also not trivial, but feasible. The use of alkylating agents (N-ethylmaleimide (NEM) or iodoacetamide) that inhibit SUMO proteases allows for preservation of the SUMOylated state. It is possible however, that the proteins within group SUMOylated assemblies may not be easily accessible to antibodies for immunoprecipitation, and/or that inhibition of deSUMOylation is not efficient enough. This may explain the discrepancy between the low levels of SUMOylated proteins detected by western blot, even after several-fold enrichment by immunoprecipitation, in contrast to the large number of SUMOylation events revealed by mass spectrometry. The use of N- or C-terminal fusion of SUMO to a protein to mimic its constitutively SUMOylated state has been successful ([113,114] and many other examples), provided that it will not perturb the function of the protein and not be attacked for degradation by the STUbL/proteasome system. Alternatively, state-of-the-art imaging techniques such as optogenetics [115] or SUMO photo-activation could help us to gain insights into SUMO dynamics within SUMO groups on the spindle (and elsewhere) in cellulo.

## 5. Spindle SUMOylation as Anticancer Target

Imbalance of SUMOylation and de-SUMOylation relates to development and progression of cancer and other diseases (for reviews, see [116,117]). Extensive work has been done on the role of SUMO and SUMO-targeted ubiquitylation on the DNA damage, transcription and signaling. Although the role of SUMO and STUbLs in regulation of microtubule- and spindle-dependent processes is less understood, proteomic and functional studies show that the functional principles through which SUMO and STUbLs regulate DNA repair and transcription apply also to the regulation of microtubule-dependent processes. Exactly this dual role of SUMO and STUbLs makes them excellent potential targets for development of anticancer drugs. In support of this idea, combinations of both MT-targeting and DNA-damaging agents are preferred for the treatment of many solid cancer forms and lymphomas [118]. Moreover, some successful, already approved anticancer tubulin-destabilizing agents like colchicine, methiazole and parbendazole have been reported to induce also DNA damage [119]. This evidence supports the idea that affecting both tubulin and DNA functions may render anticancer therapy more efficient.

A few small molecules inhibiting SUMOylation could be considered for the treatment of cancer [120], where several proteins of the SUMOylation machinery are found to be overexpressed [50]. As inhibitors of the E1 enzyme SAE1/SAE2, one finds ginkgolic acid [121], anacardic acid [121], kerriamycin B [122], davidiin [123], or the more recent COH000 [124], while 2-D08 and spectinomycin are targeting the Ubc9 enzyme [125,126]. Promising results have been obtained in cellulo and in xenograft mouse models [127,128], but none of these molecules made it yet to clinical trials (https://clinicaltrials.gov/). However, a recently developed derivative of the SUMO E1 inhibitor ML-792 [129] is being currently tested in a phase 1 clinical trial in patients with metastatic solid tumors and lymphomas (G. Bossis, personal communication).

The clearest example for the potential of SUMO/STUbLs as drug targets is the mechanism of action of arsenic trioxide during treatment of Acute Promyelocytic Leucemia (APL) cells [130]. Here, arsenic oxide leads to activation of SUMO-targeted ubiquitylation of the PML-RARα oncoprotein by RNF4, resulting in its degradation of the PML-RARα fusion protein by the proteasome. In combination with a retinoic acid, that both activates RNF4 and differentiates the immature myeloma cells, AML can be cured with a rate of 90%.

## 6. Concluding Remarks

Recent advances in proteomics have generated a wealth of information concerning targets of SUMOylation, modification sites, and their relationship to other PTMs. These data reveal that SUMOylation is an important regulator of important factors in spindle organization, ranging from MAPs to kinetochore-associate proteins. Enforcement of protein assembly through interactions between SUMO and SIMs and the principles of group SUMOylation and phase separation could operate also during the organization of the mitotic spindle. In addition, SUMO-dependent ubiquitylation participates in disassembly of spindle complexes and quality control. Study of these processes in detail promises to produce rewarding results both in basic and applied research.

## Figures and Tables

**Table 1:** List of the SUMOylated spindle proteins for which the function of SUMOylation has been studied in the listed publications and model systems.

SUMOylated Protein Studied	Localization in Mitosis	SUMO Regulates Localization?	SUMO Regulates Enzymatic Activity?	SUMO Pathway Components Involved	Model System	Reference
**ANAPC4**	kinetochore	no	N.A.	SUMO2	Mammalian cell culture	[51,60]
**Aurora B/AURKB (AIR-2, CPC)**	chromosomes, midzone, Ring complexes	yes	N.D.	SUMO2/3, PIAS2/3/4, ULP-1 (SENP2/3/5, SENP6/7	*C. elegans* mitosis, meiosis	[61,62]
**Aurora B/AURKB (CPC)**	centromere	yes	no/yes	SUMO2/3, PIAS3, SENP2	Mammalian cell culture	[44,45]
**BIRC5** **(Survivin, Bir1, CPC)**	centromere	yes	N.A.	N.D.	*S. cerevisiae*	[35]
**BUB1B (BubR1)**	kinetochore	no	N.D.	SUMO1,2/3, SENP2	Mammalian cell culture	[36,52]
**CDCA8** **(Borealin)**	Centromere, central spindle	no	N.A.	SUMO2/3, RanBP2, RRSU, SENP3	Mammalian cell culture	[43,46]
**CENPI**	centromere/kinetochore	yes	N.A.	SENP6	Mammalian cell culture	[40,63]
**Cep3**	centromere	yes	N.A.	Siz1, Siz2	*S. cerevisiae*	[35]
**Cse4/CENPA**	centromere	yes	N.A.	Siz1, Siz2, Slx5/8	*S. cerevisiae*	[64,65]
**Kar9**	astral microtubules	yes	N.A.	Siz1, Siz2, Slx5/8	*S. cerevisiae*	[66,67]
**KIF10** **(CENP-E, kinesin-7)**	kinetochore	no	N.D.	SUMO2/3, RNF4	Mammalian cell culture	[36]
**KIF18A** **(kinesin 8)**	kinetochore	no	N.D.	SUMO2	Mammalian cell culture	[47]
**KLP-19** **(kinesin 4)**	Ring complexes	yes	N.A.	PIAS2/3/4 (GEI-17)	*C. elegans* meiosis	[68]
**KIF23/MKLP1**	midzone	N.D.	N.D.	SUMO2, RNF4	Mammalian cell culture	[69]
**MIS18BP1**	kinetochore	N.D.	N.D.	SUMO2, SENP6, RNF4	Mammalian cell culture	[69,70]
**Ndc10**	centromere	yes	N.A.	Siz1, Siz2	*S. cerevisiae*	[35]
**Ndc80**	kinetochore	no	N.A.	N.D.	*S. cerevisiae*	[35]
**NKAP**	kinetochore	no	N.A.	SUMO1, SUMO2	Mammalian cell culture	[48]
**NUF2**	kinetochore	N.D.	N.A.	SUMO2/3, SENP2	Mammalian cell culture	[36]
**NuMA**	spindle pole	not clear	N.A.	SUMO1	Mammalian cell culture	[71]
**PARP1**	chromosomes	no	no	SUMO2/3, PIAS4	*Xenopus* egg extracts	[42,54]
**PICH**	centromere	no	likely	SUMO2/3, PIAS4	*Xenopus* egg extracts	[42]
**PLK1**	kinetochore, midbody	N.D.	N.D.	N.D.	Drosophila	[72]
**PLK1**	kinetochore	N.D.	N.D.	SUMO2/3	Mouse oocyte (meiosis)	[73]
**PLK1**	spindle pole	N.D.	N.D.	SUMO1	Mouse oocyte (meiosis)	[73]
**RANGAP1**	kinetochore, microtubules	yes	N.A.	SUMO1	Mammalian cell culture	[29,49,74]
**SEPT2**	spindle, midbody	N.D.	N.A.	SUMO1	Mouse oocyte (meiosis)	[75]
**SEPT3, SEPT6, SEPT7, SEPT9 (Septins)**	cleavage furrow, actin	yes	N.A.	SUMO1, SUMO2	Mammalian cell culture	[76]
**Stu2/XMAP215, TOG)**	kinetochore, microtubules	N.D.	N.A.	Smt3, Siz1, Siz2	*S. cerevisiae*	[77]
**Topoisom. II/TOP2**	chromosomes	N.D.	yes	SUMO2/3, PIAS4	*Xenopus* egg extracts	[55,56]
**Topoisom. II/TOP2**	chromosomes	N.D.	N.D.	RanBP2, SENP3	Mouse Embryonic Fibroblasts, Mammalian cell culture	[59,78]
**Topoisom. II/Top2**	chromosomes	N.D.	N.D.	Siz1, Siz2	*S. cerevisiae*	[57,58]
**TTK (Mps1)**	kinetochore	no	N.D.	SUMO1, SUMO2	Mammalian cell culture	[53]
**N.A.**	Not Applicable					
**N.D.**	Not determined					

**Table 2:** Examples of interesting SUMOylated spindle proteins from the Supplementary Table 2 in [34]. These include proteins from Table 1, supplemented with a non-exhaustive list of mitotic proteins.

SUMOylated Protein	Nr of SUMO Sites	Localization in Mitosis	Studied (Table 1)
**ANAPC4**	2	kinetochore	yes
**AURKA (Aurora A)**	9	spindle pole	
**AURKB (Aurora B)**	13	centromere	yes
**BIRC5 (Survivin)**	7	centromere	yes
**BUB1**	9	kinetochore	
**BUB1B**	1	kinetochore	yes
**BUB3**	12	kinetochore	
**CASC5 (KNL1)**	87	kinetochore	
**CDCA8 (Borealin)**	7	centromere	yes
**CENPB**	5	centromere	
**CENPC (Mif2)**	28	centromere/kinetochore	
**CENPH**	7	centromere/kinetochore	
**CENPI**	1	centromere/kinetochore	yes
**CENPK**	2	centromere/kinetochore	
**CENPN**	6	centromere/kinetochore	
**CENPO**	7	centromere/kinetochore	
**CENPQ**	8	centromere/kinetochore	
**CENPT**	2	centromere/kinetochore	
**CENPU**	11	centromere/kinetochore	
**CENPV**	3	centromere/kinetochore	
**CENPW**	1	centromere/kinetochore	
**INCENP**	10	centromere	
**KIF11 (Cin8, Eg5)**	2	microtubule	
**KIF13A**	1	microtubule	
**KIF14**	1	spindle pole, microtubule	
**KIF15**	2	microtubule	
**KIF18A**	32	kinetochore	yes
**KIF18B**	7	microtubule	
**KIF22**	24	chromosome	
**KIF23**	1/31	spindle midzone (from anaphase)	yes
**KIF2A**	1/5	centromere	
**KIF2C**	1/16	centromere	
**KIF4A**	22	chromosome	yes
**KIFC1 (kin-14)**	6	microtubule	
**KIFC3**	1	microtubule	
**MAP9**	1	microtubule	
**MIS18A**	5	centromere	
**MIS18BP1 (KNL2)**	80	centromere	yes
**NDC80**	17	kinetochore	yes
**NKAP**	5	kinetochore	yes
**NUF2**	6	kinetochore	yes
**NuMA1**	10	spindle pole	yes
**PARP1**	48	chromosomes	yes
**PLK1**	5	centromere/kinetochore, spindle pole	yes
**RANBP1**	2	kinetochore	
**RANBP2**	48	kinetochore	
**RANGAP1**	9	kinetochore, microtubule	yes
**RCC1**	8	chromosome	
**RCC2 (TD-60)**	12	centromere/kinetochore	
**SPC25**	5	kinetochore	
**TOP1**	25	chromosome	
**TOP2A**	85	chromosome	yes
**TOP2B**	2/75	chromosome	yes
**TPX2**	39	spindle pole, microtubule	
**TTK (Mps1)**	16	kinetochore	yes
